# The Utilization of Plant-Material-Loaded Vesicular Drug Delivery Systems in the Management of Pulmonary Diseases

**DOI:** 10.3390/cimb45120624

**Published:** 2023-12-12

**Authors:** Bongani Sannyboy Lukhele, Kokoette Bassey, Bwalya Angel Witika

**Affiliations:** Department of Pharmaceutical Sciences, School of Pharmacy, Sefako Makgatho Health Sciences University, Pretoria 0204, South Africa; bongani.sannyboy@gmail.com

**Keywords:** plant bioactives, vesicular drug delivery system, chronic obsessive pulmonary disorder, asthma, tuberculosis

## Abstract

Medicinal plants have been utilized to treat a variety of conditions on account of the bioactive properties that they contain. Most bioactive constituents from plants are of limited effectiveness, due to poor solubility, limited permeability, first-pass metabolism, efflux transporters, chemical instability, and food–drug interactions However, when combined with vesicular drug delivery systems (VDDS), herbal medicines can be delivered at a predetermined rate and can exhibit site-specific action. Vesicular drug delivery systems are novel pharmaceutical formulations that make use of vesicles as a means of encapsulating and transporting drugs to various locations within the body; they are a cutting-edge method of medication delivery that combats the drawbacks of conventional drug delivery methods. Drug delivery systems offer promising strategies to overcome the bioavailability limitations of bioactive phytochemicals. By improving their solubility, protecting them from degradation, enabling targeted delivery, and facilitating controlled release, drug delivery systems can enhance the therapeutic efficacy of phytochemicals and unlock their full potential in various health conditions. This review explores and collates the application of plant-based VDDS with the potential to exhibit protective effects against lung function loss in the interest of innovative and effective treatment and management of respiratory illnesses.

## 1. Introduction

There has been an increase in the number of respiratory diseases afflicting human beings [1]. Among the most prevalent are asthma, tuberculosis, chronic obstructive pulmonary disease, lung cancer, and recently, the coronavirus disease 2019 (COVID-19) [2,3]. Many of these ailments result in aberrant physiological functioning of the respiratory system and can develop as a result of a weakened immune system, smoking, microbial infection, genetics, and environmental factors including pollution [4].

Chronic obstructive pulmonary disease (COPD) and asthma are the two most prevalent pulmonary disorders, which affect the lungs’ airways and other components, including the nasal cavities, the pharynx, the larynx, the trachea, the bronchi and bronchioles, the tissues of the lungs, and the respiratory muscles of the chest cage [5,6,7]. About 3 million people died from chronic obstructive pulmonary disease (COPD), the third most common cause of mortality in the world in 2019 [8].

**Figure 1:**
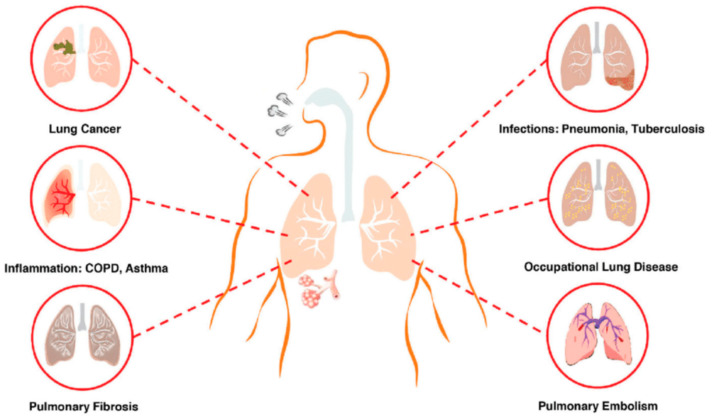
Common respiratory diseases: respiratory diseases among the leading cause of deaths worldwide. Obtained and reproduced with permission from Ref. [18], 2020, Taylor and Francis, Ltd.

In low- and middle-income nations, COPD fatalities in people under 70 years of age account for about 90% of all mortality [9]. Chronic bronchitis and emphysema are the two most prevalent diseases associated with COPD [10]. More than 5% of people in industrialized nations suffer with asthma, although it is not properly recognized or treated [11]. Asthma affects people of all ages and is one of the most common chronic diseases worldwide [12,13]. Approximately 9.8% of adult females and 6.1% of adult males have asthma. It is the most prevalent chronic illness in children. About 5.1 million children under the age of 18 suffer with asthma at the moment [14]. Among a plethora of respiratory illnesses, tuberculosis remains among the leading infectious causes of death worldwide, continuing to be a huge global health issue [15,16]. Globally, 1.6 million individuals died from TB in 2021, while 10.6 million people were diagnosed with the disease, including 3.6 million women, 1.2 million children, and 6 million males [17]. A brief synopsis of ailments that primarily affect the respiratory system is provided in Figure 1.

The World Health Organization (WHO) encourages the use herbal medicine and pharmacological research to improve the use of herbal remedies in the treatment of various respiratory disorders [19]. 

Plant materials have long been used in traditional medicine and as precursors for the development of conventional medicines. Most bioactive constituents of phytomedicines are limited in their effectiveness due to their poor systemic absorption when administered orally or topically. The limited effectiveness of bioactive constituents of phytomedicines in terms of poor systemic absorption can be attributed to several factors, viz., poor bioavailability, first-pass metabolism, poor lipid solubility, efflux transporters, and chemical instability [20,21]. Moreover, traditional dosage forms, viz., herbal decoctions and infusions, inhalations and steam therapy, herbal powders and pastes, syrups and honey-based preparations used to treat a variety of respiratory illnesses also have reduced bioavailability and less therapeutic efficacy [22,23,24]. As a result, most plant-derived actives undergo acidic degradation in the gastrointestinal tract, making them poor candidates for oral administration [25].

**Figure 2:**
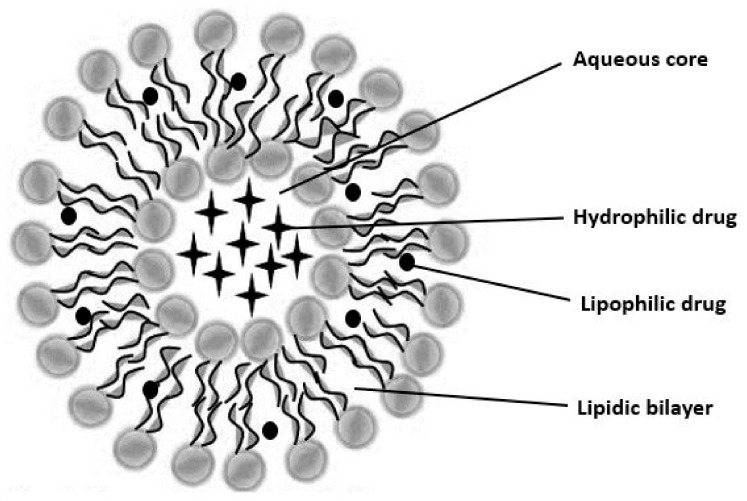
The general structure of a vesicular drug delivery system.

In recent times, there has been a concerted effort to develop vesicular drug delivery systems (VDDS) capable of delivering hydrophilic and hydrophobic materials while achieving clinically relevant concentrations. Vesicular drug delivery systems are innovative formulations that utilize vesicles to encapsulate and transport drugs to specific targets in the body. Liposomes and niosomes are two of the most prominent types of vesicles used in drug delivery. The general structure of a VDDS is provided in Figure 2.

These vesicular drug delivery systems offer numerous benefits, including enhanced drug stability, solubility, and absorption. They can also facilitate targeted delivery, reducing side effects and improving therapeutic outcomes [26,27]. The use of VDDS has the potential to circumvent many of the challenges of conventional drug delivery, such as poor absorption, toxic effects, and off-target drug delivery [28,29]. Conjugating and/or adsorbing and/or encapsulating of bioactive phytoconstituents into VDDS has the potential for spatiotemporal payload while also addressing the barriers of poor lipophilicity [30,31].

In this article, we explore and present a comprehensive discussion on the use of plant-based vesicular drug delivery technologies for the management of pulmonary illnesses.

## 2. Conventional Herbal Application in the Treatment of Pulmonary Diseases

A notable trend in medicine is the use of medicinal plants to cure ailments and improve general health [32]. Using a wide variety of plant materials for both therapeutic and preventative reasons, ‘phytomedicine’ refers to an alternative medicine approach centered on plant-based therapies. Due to their natural abundance of active ingredients, medications made from plants and herbs may provide patients with a range of health advantages [33].

Conventional medicine plays a role in treating conditions such as pulmonary diseases and respiratory infections, but there is a growing interest in complementary and alternative therapies. Among these alternative approaches, herbal medicine has a long history of use in addressing pulmonary ailments [34]. Examples of such pulmonary diseases and conventional herbal therapy include, but not limited to:

### 2.1. Chronic Obstructive Pulmonary Disorder (COPD)

The Global Initiative for Chronic Obstructive Lung Disease (GOLD) 2023 report defines COPD as a heterogeneous lung condition characterized by chronic respiratory symptoms (dyspnea, cough, expectoration, exacerbations) due to abnormalities of the airways (bronchitis, bronchiolitis) and/or alveoli (emphysema) that cause persistent, often progressive, airflow obstruction. According to the GOLD 2023 report, COPD has risen to become one of the top three leading causes of mortality worldwide, with a staggering 90% of these fatalities occurring within low- and middle-income countries (LMIC) [35]. LMICs frequently encounter elevated rates of risk factors associated with Chronic Obstructive Pulmonary Disease (COPD), including tobacco smoking, indoor air pollution arising from the use of solid fuels, and occupational exposure to pollutants. The provision of sufficient healthcare infrastructure and services poses challenges in many LMICs. The restricted accessibility to healthcare facilities can contribute to delayed diagnosis and suboptimal management of respiratory conditions, thereby advancing the progression of COPD [36].

Two disorders, chronic bronchitis and emphysema, are linked to COPD. COPD is brought on by prolonged inhalation of irritants and toxins, which leads to chronic inflammation in the airways and destruction to the alveolar structures of the lungs [37,38,39]. An abnormal enlargement of the air spaces distal to the terminal, non-respiratory bronchiole, along with damaging alterations to the alveolar walls, are the hallmarks of the lung disorder known as pulmonary emphysema [40]. The management of COPD primarily involves bronchodilators, anti-inflammatory drugs, and other conventional therapies. However, certain phytochemicals found in plants have been studied for their potential in COPD management.

Curcumin is a polyphenol derived from the rhizomes of turmeric (*Curcuma longa*). Curcumin exhibits various biological activities that may be beneficial in COPD management [41]. Curcumin has demonstrated anti-inflammatory effects by inhibiting multiple inflammatory pathways and reducing the production of pro-inflammatory mediators. It also exhibits antioxidant properties, which may help mitigate oxidative stress in the lungs [42].

Quercetin is a flavonoid widely distributed in fruits, vegetables, and medicinal plants. It has a chemical structure characterized by the presence of multiple hydroxyl groups [43]. Quercetin has antioxidant characteristics in addition to anti-inflammatory activities, which help with the management of COPD. This is done by altering several inflammatory signaling pathways and preventing the release of pro-inflammatory mediators [44].

Resveratrol is a stilbenoid compound found in grapes, berries, and other plant sources. It possesses a stilbene backbone with hydroxyl groups [45]. By blocking inflammatory mediators and altering the signaling pathways involved in inflammation, resveratrol has anti-inflammatory actions. Additionally, it has antioxidant qualities that may lessen oxidative stress in the lungs. Resveratrol also has anti-fibrotic capabilities that are important for the treatment of COPD [46].

Gingerol is a bioactive compound found in ginger (*Zingiber officinale*). It belongs to the class of phenolic compounds and exhibits various biological activities [47]. Gingerol has antioxidant characteristics and has bronchodilatory effects, both of which are important for the management of COPD. Gingerol also has anti-inflammatory actions which reduce the generation of pro-inflammatory mediators and modify inflammatory signaling pathways [48].

Ginsenosides are triterpene saponins found in *Panax ginseng*. They possess a complex chemical structure and exhibit diverse biological activities [49]. By altering inflammatory pathways and limiting the release of pro-inflammatory mediators, ginsenosides have anti-inflammatory actions. They also have antioxidant qualities and may control immunological responses, both of which are important for managing COPD [50].

### 2.2. Tuberculosis

Developing nations, particularly those in Asia and Sub-Saharan Africa, are understandably concerned about the spread of tuberculosis (TB) because they lack the resources necessary to effectively manage it. While there is ongoing research on the utilization of phytochemicals in drug delivery systems for the treatment of tuberculosis (TB), most studies focus on the use of conventional anti-TB drugs. The management of tuberculosis (TB) primarily relies on anti-tuberculosis drugs. However, there are several phytochemicals found in plants that have been studied for their potential in supporting TB management.

Allicin is a sulfur-containing compound found in garlic (*Allium sativum*). It is formed when garlic is crushed or chopped [51]. Crushing or chopping garlic disrupts cell compartments, allowing alliin and alliinase to interact. A Maillard reaction occurs. In the Maillard process, alliin and alliinase rapidly create allicin. Allicin is formed by atom rearrangement and sulfur-containing-compound release [52]. Allicin exhibits antimycobacterial activity by disrupting the cell wall of *Mycobacterium tuberculosis* and inhibiting bacterial growth. It possesses immunomodulatory effects by stimulating the production of cytokines and enhancing macrophage function [53].

Quercetin exhibits antimycobacterial activity against *Mycobacterium tuberculosis* by inhibiting bacterial growth and suppressing the formation of biofilms [43]. Quercetin also displays immunomodulatory effects by modulating immune responses, such as enhancing the production of nitric oxide and pro-inflammatory cytokines involved in immune defense [54].

Curcumin exhibits antimycobacterial activity against *Mycobacterium tuberculosis* by inhibiting bacterial growth and suppressing biofilm formation [55]. Curcumin possesses immunomodulatory properties by modulating the production of cytokines, enhancing macrophage activity, and promoting the differentiation of T cells, which are crucial for an effective immune response [56].

Embelin is a natural compound derived from the plant *Embelia ribes*. It exhibits antimycobacterial activity against drug-resistant strains of *Mycobacterium tuberculosis* by disrupting bacterial cell membranes and inhibiting bacterial growth [57]. Embelin displays immunomodulatory effects by enhancing the production of pro-inflammatory cytokines and promoting the phagocytic activity of macrophages, which contribute to host defense [58].

Berberine is an isoquinoline alkaloid found in various plants, including *Berberis* species. It exhibits antimycobacterial activity against *Mycobacterium tuberculosis* by inhibiting bacterial growth and disrupting bacterial membranes [59]. Berberine also possesses immunomodulatory properties by enhancing the production of pro-inflammatory cytokines and activating the innate immune response [60].

### 2.3. Asthma

The Global Initiative for Asthma (GINA) defines asthma as a history of respiratory symptoms including wheezing or repetitive coughing, dyspnea, and chest tightness plus variable expiratory airflow limitation, all of which vary over time and in intensity [61]. The exact causes of asthma are not fully understood, but it is thought to be caused by a combination of genetic and environmental factors [62,63].

Allergens, air pollution, exercise, and respiratory infections can trigger asthma symptoms in different people. Bronchodilators and inhaled corticosteroids open airways and reduce inflammation to treat asthma. Asthma can be fatal if untreated. Thus, early diagnosis and management improve outcomes and reduce complications [64]. The management of asthma primarily focuses on bronchodilators, anti-inflammatory drugs, and other conventional treatments. However, there are certain phytochemicals found in plants that have been investigated for their potential in asthma management.

Quercetin exhibits anti-inflammatory effects by inhibiting the release of pro-inflammatory mediators and modulating inflammatory signaling pathways [65]. Quercetin displays bronchodilatory properties by relaxing the smooth muscles of the airways and has been shown to modulate immune responses and reduce airway hyperresponsiveness in asthma [66]. 

Curcumin possesses anti-inflammatory effects by inhibiting the activation of inflammatory cells and suppressing the production of pro-inflammatory mediators [42]. It has been demonstrated that curcumin reduces airway inflammation and improves lung function in asthma experimental models. Curcumin exhibits antioxidant properties that reduce oxidative stress in the airways [67]. 

Boswellic acids are pentacyclic triterpenes found in the resin of *Boswellia serrata* [68]. Boswellic acid plays a role in asthma treatment by exhibiting anti-inflammatory actions resulting in preventing the production of cytokines and enzymes that cause inflammation. Boswellic acids have been demonstrated to decrease airway inflammation, block bronchoconstriction, and thereby enhance lung function [69].

Gingerol exhibits anti-inflammatory effects by inhibiting the production of pro-inflammatory mediators and modulating inflammatory signaling pathways. In investigational models of asthma, gingerol has also been found to lessen airway inflammation and enhance lung function by relaxing the smooth muscles of the airways [47].

Epigallocatechin gallate (EGCG) is a catechin found in green tea (*Camellia sinensis*) [70]. It exhibits anti-inflammatory effects by inhibiting the activation of inflammatory cells and reducing the production of pro-inflammatory mediators [71]. EGCG exhibits anti-oxidant qualities that lessen oxidative stress in the airways and has been demonstrated to enhance lung function and decrease airway inflammation in asthmatic experimental models [72]. Table 1 illustrates a summary of the structural features that make plant-derived compounds potentially advantageous in promoting and sustaining pulmonary health.

## 3. The Contribution of Various Phytochemicals to Respiratory Health

The respiratory system serves as a vital interface between the human body and its external environment, facilitating the exchange of oxygen and carbon dioxide while safeguarding against harmful agents [80]. In the context of a modern world characterized by pollution, allergens, and emerging infectious threats, maintaining optimal respiratory function is of paramount importance [81]. Phytochemicals have emerged as promising bioactive agents with diverse mechanisms of action that can positively impact respiratory well-being, as they encompass an extensive array of compounds synthesized by plants as part of their defense mechanisms and ecological interactions [82,83]. These compounds have long been recognized for their therapeutic potential in various health domains, and their role in supporting respiratory health is increasingly under the scientific spotlight [84,85]. The unique chemical structures and biological activities of phytochemicals have led to investigations into their effects on lung function, inflammation, oxidative stress, and immune modulation within the context of respiratory physiology [86,87,88].

A wide range of phytochemical classes, including alkaloids, flavonoids, lignans, saponins, and terpenoids, have demonstrated intriguing properties that could contribute to respiratory well-being [89,90]. Alkaloids, for instance, have shown bronchodilatory and anti-inflammatory effects, which are relevant to conditions such as asthma and COPD [91]. Flavonoids, with their potent antioxidant and anti-inflammatory properties, have garnered attention for their potential to mitigate oxidative stress-induced lung damage and inflammation-associated respiratory disorders [92].

Lignans, saponins, and terpenoids, while less explored in the respiratory context, exhibit promising attributes that could impact lung health. Lignans’ antioxidative potential contribute to preventing cellular damage and inflammation within the respiratory tract [93]. Saponins, known for their mucolytic and anti-inflammatory properties, have the potential to alleviate respiratory congestion and irritation [94]. Terpenoids, often found in essential oils, possess antimicrobial and expectorant qualities that could aid in managing respiratory infections and improving airway clearance [95].

The phytochemical constituents of various plant species have been investigated for their potential bioactive roles in supporting respiratory health. Notably, the aforementioned phytochemicals have demonstrated distinct properties that contribute to their beneficial effects within the respiratory system.

### 3.1. Alkaloids: Bridging Bronchodilation and Immune Modulation

**Figure 3:**
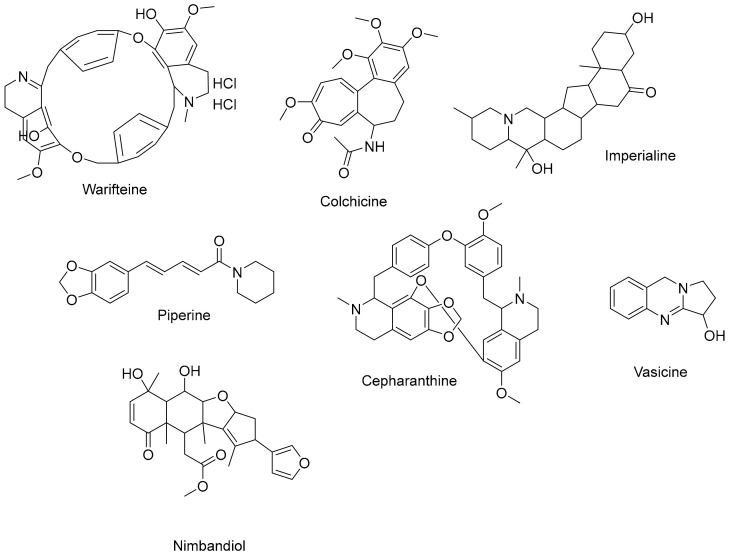
Several alkaloids that have a function in the respiratory system.

Alkaloids are naturally occurring compounds found in a wide range of plant families, including but not limited to Solanaceae (nightshades), *Papaveraceae* (poppy family), and *Ranunculaceae* (buttercup family) [96]. Alkaloids, characterized by their nitrogen-containing structures, exhibit a spectrum of pharmacological effects within the respiratory system [97]. Alkaloids contribute to bronchodilation by engaging adrenergic receptors, relaxing bronchial smooth muscles, and facilitating improved airflow. Beyond bronchodilation, alkaloids demonstrate immunomodulatory properties, influencing immune responses crucial for respiratory health [98,99]. A typical class of alkaloids is depicted in Figure 3.

### 3.2. Saponins: Surfactant Enhancement and Immunomodulation

**Figure 4:**
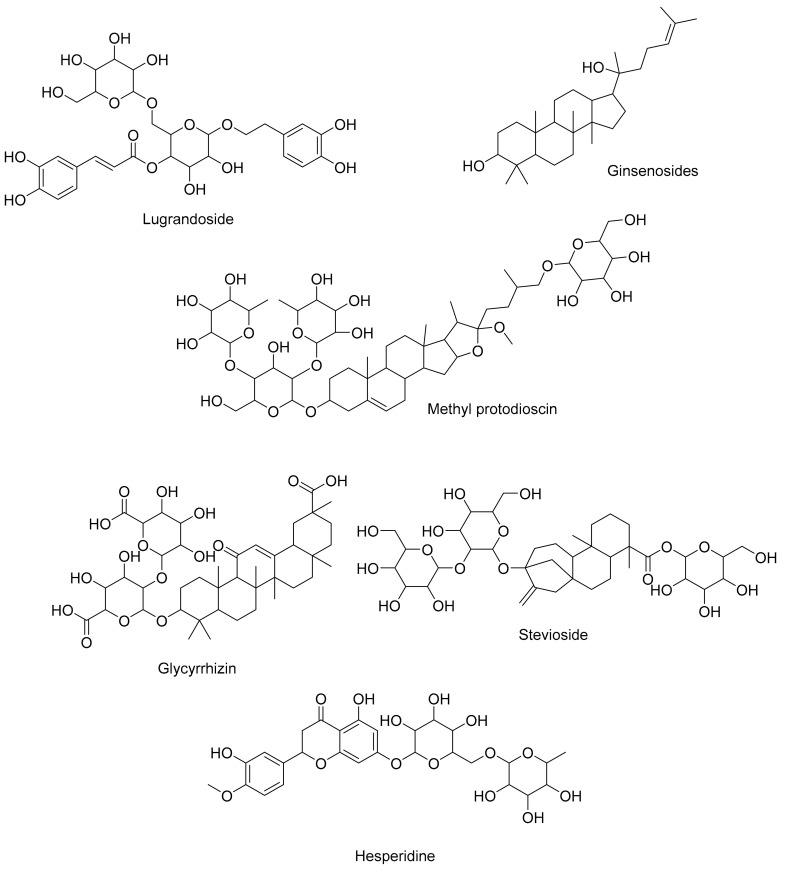
Several saponins that have a function in the respiratory system.

Saponins are present in various plant families, including *Fabaceae* (legumes), *Quillajaceae* (soapbark family), and *Asteraceae* (daisy family) [100]. Saponins, notable for their amphiphilic nature, exert diverse influences on respiratory physiology. Saponins impact mucus viscosity, enhancing mucus clearance and potentially alleviating respiratory congestion [101]. Furthermore, saponins possess immunomodulatory effects, bolstering immune cell activity and cytokine production, thereby influencing the immune response to respiratory infections [102]. The dynamic interplay between saponins and respiratory processes highlights their potential therapeutic relevance. A summary of some of the saponins that aid respiratory health are depicted in Figure 4.

### 3.3. Lignans: Hormonal and Antioxidant Potential

**Figure 5:**
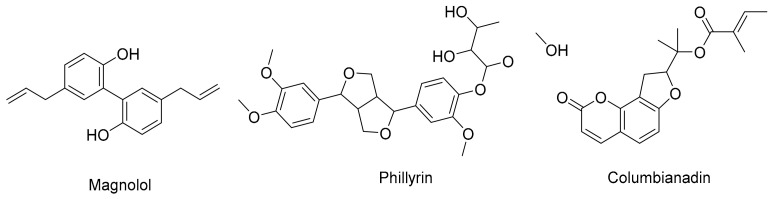
Some lignans that have a function in the respiratory system.

Lignans are present in a variety of plant families, including *Linaceae* (flax family), *Juglandaceae* (walnut family), and *Lamiaceae* (mint family) [103]. Lignans, present in diverse plant materials, contribute a unique set of attributes to respiratory health. These compounds possess hormonal activity, potentially influencing hormonal pathways that intersect with respiratory physiology [104]. Moreover, lignans exhibit antioxidant properties, scavenging free radicals and conferring cellular protection [105]. Due to their complexity, lignans can interact in a variety of complex ways with the respiratory system. Figure 5 displays some of the lignans that contribute to respiratory support. 

### 3.4. Terpenoids: Anti-Inflammatory and Antimicrobial Allies

**Figure 6:**
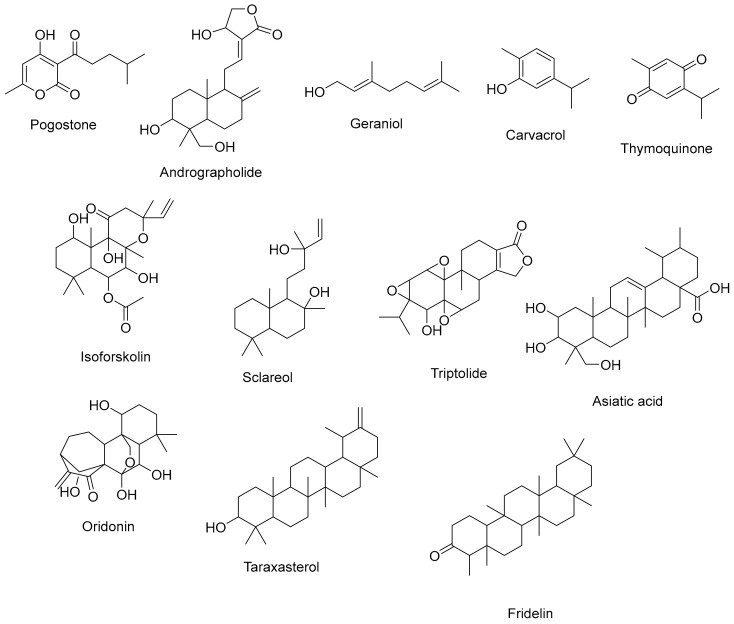
Several terpenoids that have a function in the respiratory system.

Terpenoids are found in many plant families, including *Pinaceae* (pine family), *Lamiaceae* (mint family), and *Myrtaceae* (myrtle family) [106]. Terpenoids, which are abundant in essential oils and resins, showcase a range of bioactivities pertinent to respiratory health. Their anti-inflammatory effects influence immune responses, potentially alleviating respiratory symptoms associated with inflammation [107]. Additionally, terpenoids have antibacterial activities that target respiratory pathogens and strengthen defense against infections, and their complex chemical structures highlight their potential as adaptable players in the management of respiratory health [107,108,109]. A summary of terpenoids that aid respiratory health is shown on Figure 6.

### 3.5. Flavonoids: Antioxidant Guardians and Inflammatory Modulators

**Figure 7:**
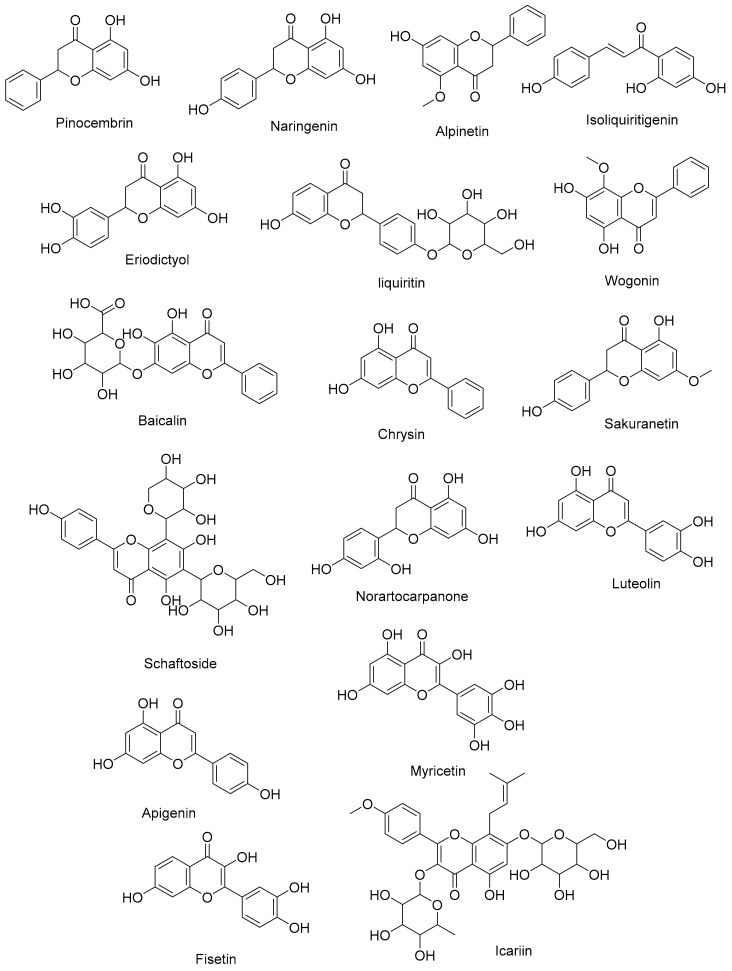
Several flavonoids that have a function in the respiratory system.

Flavonoids are widespread in plants, being present in families such as *Rosaceae* (rose family), *Rutaceae* (citrus family), and *Fabaceae* (legumes) [110]. Flavonoids, abundant in various plant sources, offer a wealth of antioxidant and anti-inflammatory properties. Within the respiratory system, flavonoids neutralize free radicals, alleviating oxidative stress and potentially mitigating cellular damage [111]. Additionally, their anti-inflammatory effects modulate cytokine production and immune cell responses, impacting respiratory inflammation [44,112]. Research into the mechanisms of oxidative stress and flavonoids offers a fascinating new direction for the study of respiratory health. Figure 7 illustrates an overview of flavonoids that promote respiratory well-being.

## 4. Physiological and Anatomical Factors Influencing Pulmonary Drug Delivery

Despite the development of potential drugs and vesicular drug delivery systems for the treatment of pulmonary disorders, there are still significant physiological and anatomical barriers that hinder effective drug delivery to the target sites within the lungs [113]. These barriers, including the mucus layer, epithelial barrier, lung clearance mechanisms, lung metabolism, vascular barrier, and respiratory disease conditions, pose challenges in achieving optimal drug concentrations at the desired locations within the pulmonary system [114]. Physiological factors play a crucial role in pulmonary drug delivery, influencing the transport and deposition of inhaled particles or aerosols in the respiratory system [115]. Understanding these factors is essential for designing effective drug delivery systems for respiratory illnesses.

### 4.1. Lung Anatomy

The respiratory system comprises the upper airways (nasal cavity, pharynx, and larynx) and the lower airways (trachea, bronchi, and bronchioles). Drug deposition and absorption primarily occur in the alveolar region, deep in the lungs. The anatomical structure of the lungs, including the branching airway network and the alveolar surface area, determines the distribution of inhaled particles or aerosols [116]. Understanding the anatomical structure of the lungs and the significance of drug distribution in the alveolar region is crucial for designing effective inhalation therapies and optimizing the therapeutic outcomes for respiratory diseases [117].

### 4.2. Mucociliary Clearance

The respiratory tract is lined with a mucus layer that serves as a defence mechanism by trapping and removing inhaled particles or pathogens. The coordinated action of cilia on the surface of epithelial cells propels the mucus toward the throat, a process known as mucociliary clearance. This mechanism can limit the residence time of drug particles in the lungs, resulting in reduced drug absorption. However, the extent of mucociliary clearance varies among individuals and can be altered in certain respiratory diseases [113,115,116].

To optimize pulmonary drug delivery, formulation strategies are approaches that are currently being exploited. Nanoparticles and microparticles can encapsulate drugs and provide controlled release profiles. They can be engineered to optimize particle size, surface properties, and drug-release kinetics, allowing for targeted delivery to specific lung regions. Liposomes, polymeric nanoparticles, and solid lipid nanoparticles are examples of delivery systems that can be formulated to encapsulate drugs and improve their stability, solubility, and targeting capabilities. These formulations can enhance drug delivery efficiency and control drug release in the lungs [118,119].

## 5. Vesicular Drug Delivery Systems (VDDS)

Vesicular drug delivery systems are specialized formulations designed to deliver drugs to specific target sites within the body. These systems use microscopic vesicles, which are small lipid-based structures, to encapsulate and deliver therapeutic agents [120]. A variety of VDDS have been developed, offering drug administration at a set pace with sustained action to boost patient compliance [4,121]. There are several types of vesicular drug delivery systems, including liposomes, phytosomes, niosomes, transfersomes, ethosomes, cubosomes, etc. [122,123,124].

Liposomes are spherical vesicles composed of a lipid bilayer that encapsulates drugs. They are biocompatible and biodegradable and can be used to deliver both hydrophilic and hydrophobic drugs. Liposomes can be surface-modified with targeting ligands to enhance their specificity for certain tissues or organs [26,125,126,127].

Niosomes are liposomes composed of non-ionic surfactants instead of phospholipids. They can encapsulate hydrophilic and hydrophobic drugs and be surface-modified for targeted drug delivery [128]. Pharmacosomes are also forms of liposomes where the drug is covalently bound to a lipid anchor, enhancing drug stability, bioavailability, and therapeutic efficacy [129,130]. Transfersomes are elastic vesicles that can deliver drugs through skin or mucosal membranes. Phospholipids and surfactants encapsulate hydrophilic and hydrophobic drugs. Ethosomes are soft vesicles composed of phospholipids, alcohol, and water. They are primarily used for transdermal drug delivery, allowing enhanced penetration into the skin layers [131]. Cubosomes are a type of nanostructured lipid-based delivery system with assembled structures composed of lipid molecules that arrange themselves in a unique cubic lattice pattern. Cubosomes encapsulate hydrophilic and hydrophobic drugs. Their high surface-to-volume ratio allows rapid drug release. Surface modification allows targeted drug delivery [132].

Phytosomes are vesicular drug delivery systems that consist of complexes formed by conjugating phytoconstituents with phospholipids. This enhances the solubility and bioavailability of plant active material, making them more effective as therapeutic agents [31]. Phytosomes share similarities with other drug delivery systems; their unique characteristic of incorporating plant extracts distinguishes them as a specialized formulation for enhancing the delivery of phytoconstituents with potential therapeutic benefits [26,128,131].

**Figure 8:**
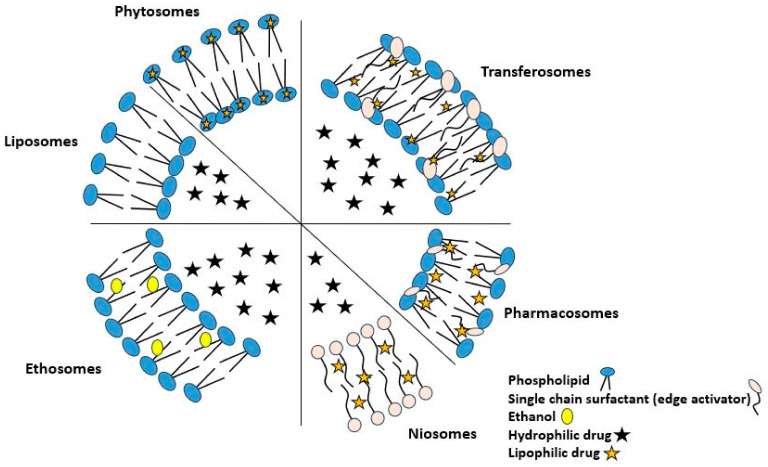
Diagrammatic representation of different types of vesicular drug delivery systems.

All drug delivery systems aim to improve drug delivery and enhance therapeutic efficacy. While they share some common goals and principles, they differ in composition, drug encapsulation mechanisms, targeting strategies, and release properties. The selection of a particular system depends on the specific requirements and characteristics of the drug being delivered and the desired therapeutic outcome [125,133]. Structures and a summary of the properties of the aforementioned VDDS are provided in Figure 8 and Table 2, respectively.

## 6. General Preparation and Characterization Techniques of Plant-Material-Loaded VDDS

Vesicular drug delivery systems that are encapsulated with plant material offer unique advantages for the targeted and controlled delivery of therapeutic agents. Several vesicular systems, including liposomes, phytosomes, and niosomes, have been explored for encapsulating plant materials and delivering them to specific sites in the body. Vesicular drug delivery systems can be prepared using various methods, which can be broadly categorized into active loading and passive loading methods. These methods are chosen based on the nature of the drug payload, whether it is hydrophilic or hydrophobic.

Phytosomes can be classified based on their lamellarity, encompassing Small Unilamellar Vesicles (SUV), Large Unilamellar Vesicles (LUV), or Multi-Lamellar Vesicles (MLV). The optimization of specific delivery needs may require various sizes of phytosomes, and diverse methods are utilized to create these nanocarrier systems. The preparation of phytosomes can vary depending on the intended vesicle size, size distribution, number of bilayers, desired entrapment efficiency of the aqueous phase, and the required permeability of the vesicle membranes.

Different methods are utilized to achieve these specific characteristics. In general, the manufacture of phytosomes involves the combination of plant-derived bioactive compounds with phospholipids, typically phosphatidylcholine, to enhance their bioavailability. Initially, the selected plant extract and phospholipid are mixed, and the resulting blend is dissolved in an organic solvent. The solvent is then evaporated to form a thin lipid film, which is rehydrated with an aqueous solution, creating a lipid bilayer that encapsulates the plant-derived compounds. The mixture undergoes sonication or homogenization to generate small vesicles [155,159].

### 6.1. Preparation of Multilamellar Vesicles (MLV)

#### 6.1.1. Solvent Evaporation

The method involves adding both phytoconstituents and phospholipid complexes to an organic solvent (methanol or ethanol)-containing flask. Phytosomes are entrapped with maximum drugs by maintaining an optimal temperature above glass transition temperature for 1 h. The organic solvent is removed using a rotary evaporator and the thin film stored overnight in desiccators after sieving with 100 mesh sieves; this will then be followed by hydration, which leads to the successful formation of phospholipid complexes [160,161]. Stability is achieved by keeping the phytosomes at room temperature in an amber-colored bottle flushed with nitrogen [162].

Damle and Mallya used solvent evaporation to prepare phytosomes; due to its high phytosome yield and entrapment efficiency, this approach was chosen for preparation. Mitomycin C-soybean (MMC)-loaded phytosomes were also synthesized by Hou et al., and a successful innovative formulation of the MMC-loaded phytosomes was established with superior formulation features such as smaller size, reduced size distribution, and better stability [163,164]. Nanosized ethosomal vesicles were formulated, which encapsulated and ensured sustained delivery of the metronidazole molecules through the regenerated cellulose semipermeable membrane. The metronidazole ethosome was made by the solvent evaporation approach [165].

#### 6.1.2. Transmembrane pH Gradient Drug Uptake

This method is particularly advantageous for ionizable hydrophobic payloads. In this process, a mixture of the hydrophobic payload, surfactant, and cholesterol is dissolved in chloroform. The solvent is subsequently evaporated under reduced pressure, forming a thin film on the inner surface of a round-bottomed flask. This film is then hydrated using a 300 mM citric acid solution at pH 4.0 with the assistance of a vortex mixer. The resulting multilamellar vesicles undergo three cycles of freezing and thawing, followed by sonication. The phytosome suspension is further combined with an aqueous solution containing the drug and vortexed. Adjusting the sample’s pH to a range between 7.0 and 7.2 is achieved by adding a 1 M disodium phosphate solution, and the mixture is heated at 60 °C for 10 min to generate multilamellar vesicles [166].

The vesicles’ neutral outer surface leads to the coexistence of both protonated and unprotonated forms of the payload. The unprotonated form, being permeable, has a tendency to cross the phytosome membrane. Within the acidic contents of the vesicle, the active pharmaceutical ingredient (API) undergoes protonation and becomes confined within the vesicle. The process of diffusion persists until there is an equilibrium in API concentration between the interior and exterior of the vesicle membrane [167].

### 6.2. Preparation of Small Unilamellar Vesicles (SUV)

#### 6.2.1. Microfluidics

Microfluidic techniques encompass innovative preparation methods utilizing microchannels with diameters ranging from 5 µm to 500 µm for precise control of material flow. A fluidized stream of lipids in ethanol or isopropanol flows through meticulously defined microchannels, engaging with an aqueous stream at ultra-high velocities within an interaction chamber. Continuous axial mixing of the two phases takes place, and phytosomes are generated through the local diffusion of surfactant molecules that self-assemble upon contact with the aqueous phase. Phytosomes produced using this technology demonstrate remarkable reproducibility and uniformity, forming vesicles of small size (<150 nm) and very low polydispersity index (<0.200) [168,169,170].

#### 6.2.2. Sonication

In the sonication method, a drug-containing aqueous phase is introduced to a blend of surfactant and cholesterol in a scintillation vial [128]. Subsequently, the mixture undergoes homogenization using a sonic probe at 60 °C for a duration of up to 3 min. The resulting vesicles exhibit small and uniform sizes. Sonication is carried out at temperatures surpassing the phase transition temperature and for a duration exceeding 20 min.

### 6.3. Preparation of Large Unilamellar Vesicles

#### 6.3.1. Solvent Ether-Injection Process

In this preparation method, phytoconstituents in an aqueous medium are treated dropwise with phospholipids that have solubilized in diethyl ether, this is followed by subsequent removal of solvents, this result in the formation of cellular vesicles thus leading to complex formation [171]. 

#### 6.3.2. Reversed-Phase Evaporation (REV)

The REV method for phytosome preparation is particularly advantageous for encapsulating hydrophilic payloads, ensuring a more pronounced aqueous core compared with the film hydration method. In this process, cholesterol and surfactant are combined in a mixture of ether and chloroform. Subsequently, an aqueous solution containing the payload is introduced, and the resulting two phases undergo sonication for 5 min at 4–5 °C. The resulting clear gel is further sonicated after the addition of approximately 10 mL of the aqueous phase. The organic phase is then removed under low pressure at ~40 °C using a rotary evaporator. The resulting viscous suspension is diluted with an aqueous phase and heated in a water bath at temperatures above the critical temperature (Tc) for 10 min, ultimately yielding phytosomes [154,172]

The primary limitation of this method is the potential existence of residual solvent, which could lead to unintended biological outcomes.

### 6.4. Miscellaneous Techniques

#### 6.4.1. Emulsion Formation

This technique begins with the creation of an oil-in-water (o/w) emulsion by combining an organic solution of surfactant, cholesterol, and an aqueous solution of the payload [173]. Subsequently, the organic solvent is evaporated, leading to the formation of phytosomes dispersed within the aqueous phase.

#### 6.4.2. Lipid Injection

The lipid injection method avoids the use of costly organic phases, making it an environmentally friendly option. Initially, a blend of lipids and surfactant is melted and then injected into a vigorously stirred, hot aqueous solution containing the payload [128,174,175].

The preparation of vesicular drug delivery systems involves a combination of lipid-based formulation techniques and drug loading strategies. These strategies are tailored based on the desired properties of the vesicles and the specific requirements of the drug being delivered. The goal is to formulate stable, efficient, and biocompatible vesicular systems that can encapsulate and deliver drugs to the desired target site.

### 6.5. Characterization of VDDS

Characterization of VDDS involves a range of techniques and parameters to evaluate their physicochemical properties, stability, drug loading, release behavior, and interactions with biological systems. A variety of methods and techniques are used to characterize VDDS, including melting point determination, thin layer chromatography (TLC), infrared spectroscopy, nuclear magnetic resonance (NMR), differential scanning calorimetry (DSC), Thermogravimetric Analysis (TGA), Differential Thermal Analysis (DTA), X-ray diffraction analysis (XRD), scanning electron microscopy (SEM), transmission electron microscopy (TEM), photon correlation spectroscopy (PCS), and drug entrapment efficiency (%EE) [176]. Characterization of VDDS also involves assessing key factors such as bilayer rigidity, lamellarity, and surface elemental composition. Bilayer rigidity impacts system stability and drug release kinetics, with techniques like AFM and FRAP used for assessment. Lamellarity, or the number of lipid bilayers, affects drug encapsulation and release; it can be visualized using Cryo-EM and TEM. Surface elemental composition is analyzed using XPS, confirming surface modifications and functionalization. SEM-EDX combines microscopy and elemental analysis to provide information about the vesicle’s surface morphology and composition, aiding in comprehensive characterization [177,178].

#### 6.5.1. Particle Size, Particle Shape, and Particle Size Distribution

##### Dynamic Light Scattering

Particle size (PS), polydispersity index (PDI), and zeta potential are important parameters for the characterization of vesicular delivery systems. These parameters provide valuable information about the size and size distribution of vesicles [179,180]. The specific ranges of these parameters may vary depending on the type of drug, target application, and the intended purpose of the drug delivery system. However, there are general guidelines regarding what constitutes a successful drug delivery system in terms of these parameters:

Particle size (PS) is an important parameter in the approximation of the success of a VDDS. VDDS for use in pulmonary diseases often have a specific target range for PS, which typically falls within the nanometer range (<1000 nm). However, for many applications, VDDS with less than 100 nm are preferred due to their increased surface area and enhanced bioavailability [181,182].

A low PDI value is desirable as it allows scientists to predict that the technology will behave the same and in a reproducible manner. A PDI < 0.3 indicates a narrow size distribution and uniform particle size. A low PDI suggests that most particles in the drug delivery system are of similar size, reducing variability in drug delivery and ensuring consistent performance [183].

##### Zeta Potential (ZP)

The zeta potential, representing the overall charge present in the medium, characterizes the charge of phytosomes within emulsions. Depending on the composition of the phytosome, the zeta potential may exhibit a negative, positive, or neutral charge. This zeta potential serves as an indicator of the stability of phytosomes in a given medium, as charged particles experience sufficient repulsion to uphold stability [180,184]. 

A phytosome emulsion exhibits stability when its zeta potential exceeds or falls below the threshold of 30 mV. The electrostatic characteristics of phytosomes are assessable through various techniques such as Doppler velocimetry, zeta sizer, master size, microelectrophoresis, pH-sensitive fluorophores, high-performance capillary electrophoresis, and instruments utilizing dynamic light scattering (DLS) [180,185,186,187,188,189].

##### Scanning Electron Microscopy (SEM), Transmission Electron Microscopy (TEM), Cryo-Electron Microscopy (Cryo-EM), Atomic Force Microscopy (AFM) and Fluorescent Microscopy

SEM and TEM are powerful techniques used for the characterization of VDDS. These imaging techniques provide valuable information about the size, morphology, surface characteristics, and internal structure of vesicles [190]. SEM enables the visualization of the surface morphology and topography of the vesicles. It produces high-resolution images by scanning a focused electron beam across the sample surface and detecting the secondary electrons emitted from the surface. SEM analysis can provide insights into the shape, size distribution, surface roughness, and aggregation state of vesicles [191]. TEM allows for examination of the particle size of vesicles. It uses a high-energy electron beam transmitted through the sample, and the resulting image provides information about the morphology, lamellar structure, and encapsulated materials within the vesicles. TEM analysis can reveal the presence of lipid bilayers, lamellar structures, or other internal arrangements in vesicular systems [192]. SEM and TEM are useful techniques for the characterization of vesicular delivery systems, providing insights into their size, morphology, internal structure, and interactions with biological systems.

Cryo-Electron Microscopy (Cryo-EM) provides high-resolution structural insights into vesicular drug delivery systems, allowing visualization of vesicle ultrastructure. It offers sub-nanometer resolution for detailed examination, preserves samples in their native state by rapid freezing, and supports 3D reconstruction to study vesicle morphology [193].

Atomic Force Microscopy (AFM) offers nanoscale imaging and mechanical property measurements of vesicles. It visualizes vesicle surface topography, measures mechanical characteristics like elasticity and adhesion forces, and utilizes force spectroscopy to investigate vesicle interactions with biological membranes [194].

Fluorescent Microscopy is essential for tracking and visualizing drug-loaded vesicles in vesicular drug delivery systems. It involves labeling vesicles and drugs with fluorescent markers for real-time tracking, co-localization studies to examine vesicle interactions with cellular structures, and quantitative analysis to assess vesicle behavior in drug delivery [195]. 

#### 6.5.2. Surface Charge

Zeta potential (ZP) is an indicator of the surface of particles. A ZP value > |30 mV| is typically considered ideal for stability and long-term performance. Particles with a relatively high absolute value of ZP tend to repel each other, preventing aggregation and enhancing stability in suspension or colloidal systems [179]. As per the Derjaguin–Landau–Verwey–Overbeek (DLVO) theory, when particles possess a high absolute value of zeta potential, they undergo robust electrostatic repulsion, which effectively opposes the attractive van der Waals forces. This electrostatic repulsion serves as a deterrent to particle aggregation, thereby augmenting the stability of colloidal systems or suspensions.

#### 6.5.3. Solubility and Partition Coefficient

Solubility and partition coefficient are the most common way of expressing the lipophilicity of nanoformulations. The partition coefficient (P) is a measure of the distribution of a compound between two immiscible phases, usually octanol and water. It is often represented as the logarithm of the ratio (log P). In the context of nanoformulations, the partition coefficient ratio plays a crucial role in determining drug loading and release kinetics from nanomaterials [196]. A favorable partition coefficient ratio for a drug implies that it can be efficiently loaded into the hydrophobic core of VDDS, ensuring sustained and controlled release. This is particularly important for achieving a prolonged therapeutic effect and minimizing potential toxicity associated with burst release of drugs [197].

Several methods exist for assessing solubility and partition coefficient ratio in nanoformulations. Effective assessment methods, such as high-performance liquid chromatography (HPLC) and drug’s octanol-water partition coefficient (log P) play a pivotal role in formulating and optimizing nano-carriers.

The log P of a drug can be determined experimentally using established procedures, such as the shake-flask method, where the drug is introduced into a mixture of octanol and water, vigorously shaken, and then analyzed to calculate the logarithm of the concentration ratio in octanol and water. Alternatively, software tools and databases can provide estimated log P values for a wide range of compounds [198]. 

HPLC is widely used to evaluate drug solubility in nanoparticles. HPLC provides precise and sensitive measurements that aid in optimizing nanoparticle preparation processes and ensuring efficient drug encapsulation [199]. Computational methods like molecular dynamics simulations and quantum chemistry calculations can help predict solubility and partition coefficients, providing valuable insights during the formulation design phase [200]. 

#### 6.5.4. Encapsulation Efficiency (EE)

Encapsulation efficiency, denoted as EE percent, quantifies the proportion of phytochemicals incorporated within the phytosome. It can be expressed mathematically as Equation (1):

(1) $$\%\mathrm{EE}=\frac{\mathrm{Amount}\;\mathrm{of}\;\mathrm{API}\;\mathrm{entrapped}}{\mathrm{Total}\;\mathrm{amount}\;\mathrm{of}\;\mathrm{API}}\times 100\%$$

The process of determining encapsulation efficiency commences by eliminating free, unencapsulated phytochemicals from the phytosome emulsion through techniques like Sephadex gel column, ultracentrifugation, or dialysis (using a defined cut-off) against a buffer solution for several hours. The second step in estimating encapsulation efficiency involves breaking down the phytosome bilayer (utilizing Triton X-100, acetonitrile, methanol, and ethanol) and measuring the liberated active agent using various methods such as enzymatic assays, gel electrophoresis, fluorescence spectroscopy, and field flow fractionation chromatographic methods like HPLC, UPLC, or LC-MS [201].

#### 6.5.5. In Vitro Drug Release

Since the release profile achieved in vitro may serve as an index of the efficacy of the carrier in vivo, the drug release behavior of vesicle carriers has been the focus of intensive investigation over the recent past. The most common traditional methods used to ascertain the release rate of active substances are continuous flow, sample and separate strategies, in situ processes, and membrane diffusion strategies (dialysis, micro-dialysis, fractionallises, and reverse dialysis) [201].

#### 6.5.6. Lamellarity

Lamellarity denotes the number of lipid bilayers within phytosomes. Commonly used techniques for assessing lamellarity include electron microscopy, 31^P^ nuclear magnetic resonance, and small-angle X-ray scattering. Among these, 31^P^ NMR stands out for its precision and simplicity in lamellarity determination. However, its drawback lies in its sensitivity to experimental conditions, such as reagent concentration, vesicle type, and buffer concentration [202,203].

#### 6.5.7. Crystallinity and Thermal Stability

##### Differential Scanning Calorimetry (DSC)

DSC measures the heat flow associated with thermal transitions in a sample, providing valuable information about the thermal behavior and stability of the vesicles. DSC allows the observation of interactions by comparing transition temperatures, the frequency of new peaks, the disappearance of peaks, melting points, and relative peak areas [204,205].

The DSC thermograms revealed distinct phase transitions and melting temperatures (T_m_) for various lipids, providing insights into the phase behavior and stability of the VDDS [206]. DSC is a valuable technique for the characterization of vesicular delivery systems, providing insights into their thermal properties, phase transitions, and stability.

##### X-ray Diffraction (XRD)

XRD analysis provides valuable information about the crystalline structure, phase behavior, and degree of order within vesicular systems [207]. For instance, XRD analysis can reveal the presence of ordered lipid bilayers in liposomes and niosomes. Lipid bilayers typically exhibit characteristic diffraction peaks, which arise from the periodic packing of lipid molecules. The position, intensity, and shape of these peaks can provide insights into the lamellar structure, chain packing, and molecular organization within the vesicles [196]. In recent studies, XRD analysis has been applied to investigate the structural properties and behavior of vesicular delivery systems. 

The molecular arrangement, packing density, and interactions between the vesicular components, as well as the changes induced by formulation parameters or external stimuli can be acquired from XRD analysis. Nsairat et al. utilized XRD to characterize the structural changes and polymorphic transitions in liposomes containing different ratios of cholesterol and phospholipids [208].

#### 6.5.8. Chemical Composition

##### Fourier Transform Infrared Spectroscopy (FTIR)

FTIR provides distinct information about functional groups according to band number, position, shape, and intensity. Comparison of the spectroscopy of phospholipid complexes and mixtures can verify the formation of phytophospholipid complexes [209,210]. Ferreira et al. used FTIR spectroscopy to study the self-assembly and molecular interactions in pH-sensitive liposomes for drug delivery [211]. Badria et al. used FTIR to investigate the encapsulation and release behavior of niosomes loaded with curcumin [212]. FTIR spectroscopy is a valuable technique for the characterization of vesicular delivery systems, providing insights into their chemical composition, molecular interactions, and structural properties [209,210].

##### Raman Spectroscopy

Raman spectroscopy has emerged as a powerful and versatile analytical technique in the characterization of vesicular drug delivery systems (VDDS). Its application provides valuable insights into the molecular composition, structural features, and critical quality attributes (CQA) of vesicles. Raman spectroscopy is highly effective in identifying and distinguishing different molecular components within vesicular systems [213]. It offers a unique fingerprint based on the molecular vibrations of the chemical bonds present. This capability is crucial for confirming the presence of specific drug molecules, lipids, or other constituents in the vesicle formulation [214]. Roda et al. invesitigated Raman spectroscopy analysis of multi-functionalized liposomes; computational line shape (CLS) analysis applied to the Raman data demonstrated a high degree of synthetic reproducibility in the formulations. Additionally, it confirmed the stability of liposomes, highlighting the technique’s capability to assess the effectiveness of liposome synthesis with minimal sample quantities [215].

## 7. Plant-Based Vesicular Delivery System and Applications in Respiratory Diseases

Numerous studies have looked at how medicinal plants may be encapsulated in plant-derived lipids and polymers to increase their effectiveness in treating respiratory illnesses such lung cancer, chronic obstructive pulmonary disease (COPD), and asthma. Delivery techniques have also been used in recent years to carry plant-derived actives to the systemic circulation by making use of the lung’s innate capacity to transfer molecules into the bloodstream [126,216,217,218]. The large surface area along the pulmonary route enables quick medication absorption. The design, development, and research of different delivery techniques for the treatment of pulmonary illnesses may successfully cure lung problems such as cystic fibrosis, asthma, chronic obstructive pulmonary disease, and lung cancer [219,220,221,222].

### 7.1. Lung Cancer

In a lung cancer chemoprevention trial conducted by Mao et al., grape seed phytosomes were evaluated for their biological activities. Human lung premalignant and malignant cells were examined for phytosome effects on prostacyclin. According to the study, phytosomes were found to be effective as chemo-preventives and anti-neoplastic agents for lung cancer [223].

### 7.2. Asthma

Using a pilot study in healthy individuals with mild-moderate episodes of asthmatic attacks and rhinitis, quercetin phytosome was compared with standard management (SM). Quercefit™ (QFIT) tablets were taken in conjunction with SM or alone (control group). The combined quercetin phytosome + SM showed superior results compared with the controls, reducing day and night symptoms, reducing variability of peak expiratory flow, maintaining higher peak flow, and preventing symptoms after 30 days of treatment [224]. 

Due to the liposome phospholipids’ similarity to the surfactants found in the lung, liposomes are recognized to be safe medication carriers for pulmonary delivery, and it has been discovered that inhaling liposomes increases the amount of time that entrapped material stays in the respiratory system [225,226]. Increased therapeutic efficacy may result from liposome-mediated pulmonary drug delivery, which can prolong drug retention in the lungs while minimizing extrapulmonary adverse effects [227]. 

Studies by Turrens et al. and Padmanabhan et al. have demonstrated that liposomal encapsulation of antioxidants significantly boosts their delivery to the lung and strengthens their protective effects against tissue damage caused by oxidants [228,229]. The primary component of vitamin E, a-tocopherol, is an antioxidant that provides strong defense against lung damage brought on by exposure to oxidants such as smoke, pollution, and hyperoxia when given in large quantities over extended periods of time [230].

The following studies suggest that vesicular drug delivery systems may have potential in the management of respiratory illnesses. However, further research is needed to establish their safety and efficacy in human subjects and to develop standardized protocols for their use.

Naringenin-Loaded Phytosomes for Lung Injury Treatment:

Phytosomes encapsulating naringenin exhibited critical quality attributes, including a zeta potential (ZP) of 20.97 ± 0.55 mV, particle size (PS) of 150.8 ± 6.9 nm, and an encapsulation efficiency (%EE) of 92.1% ± 1.87%. In the context of treating lung injury, particularly pulmonary edemas, a notable outcome was observed. The dry powder inhalation of naringenin-loaded DPPC phytosomes (NPDPIs) led to a significant reduction in cytokine expression, demonstrating promising therapeutic potential in this application [231].

Gingerol-Loaded Phytosomes for Respiratory Infections:

Another set of phytosomes, this time encapsulating gingerol, demonstrated a ZP of −13.11 mV, a PS of 254.01 ± 0.05 nm, and an %EE of 86.02 ± 0.18%. In the treatment of respiratory infections, the pharmacodynamic parameters indicated sustained antibacterial action and considerable anti-inflammatory effects against both Gram-positive and Gram-negative bacteria. This suggests the potential of gingerol-loaded phytosomes in addressing respiratory infections effectively [232].

*Boswellia serrata* Phytosomes for Asthma:

Phytosomes containing *Boswellia serrata* were utilized in the treatment of asthma. While specific critical quality attributes were not mentioned, patients receiving phytosome treatment required fewer inhalations. The observed adverse events were mild to moderate, with insomnia and nausea being the only reported side effects. This implies a potential benefit in managing asthma with *Boswellia serrata* phytosomes [233].

Curcumin Liposomes for Asthma:

Liposomes loaded with curcumin exhibited a ZP of −61.0 mV, a PS of 271.3 ± 3.06 nm, and an %EE of 81.1%. In the context of asthma treatment, the curcumin liposome formulation showed efficacy in suppressing pro-inflammatory markers (IL-6, IL-8, IL-1β, and TNF-α) in the BCi-NS1.1 cell line. This suggests a potential role for curcumin liposomes in mitigating inflammation associated with asthma [234].

a-Tocopherol Liposomes for Lung Injury:

Liposomes loaded with a-tocopherol were effective in preventing oxidative lung damage. While specific critical quality attributes are not detailed, the formulation demonstrated high efficacy in protecting against lung injury associated with oxidative stress. This highlights the potential therapeutic value of liposomal a-tocopherol in lung injury scenarios [235].

Paclitaxel Liposomes for Lung Cancer:

In the context of lung cancer treatment, liposomes loaded with paclitaxel showcased a decreased number of observable tumor foci on lung surfaces. The treatment also led to increased medication effectiveness and longer survival, indicating the potential of paclitaxel liposomes in improving outcomes for lung cancer patients [236].

Table 3 provides a summary of the significant work done in the field of plant-based VDDS in the management of respiratory disorders.

## 8. Conclusions and Future Perspective

Respiratory illnesses, such as asthma or chronic obstructive pulmonary disease (COPD), often require multiple medications for effective treatment. Drug vesicular systems offer the potential for co-encapsulation of multiple drugs within a single carrier, allowing for combination therapy. This approach can improve treatment outcomes by delivering multiple medications simultaneously, enhancing synergistic effects, and reducing the number of separate inhaler devices or nebulizers needed. With advances in gene and RNA-based therapies, drug vesicular systems can facilitate the delivery of genetic material to target cells in the lungs. VDDS can protect fragile nucleic acids from degradation and assist in their uptake by cells. This opens new possibilities for treating genetic respiratory disorders or utilizing gene-editing techniques to correct underlying genetic abnormalities. In the future, drug vesicular systems can be tailored to individual patients based on their specific respiratory conditions and genetic profiles. Advances in nanotechnology and precision medicine may enable the development of customized vesicular carriers that optimize drug delivery for each patient, improving treatment efficacy and minimizing side effects.

Applications of VDDS in the pharmaceutical industry will expose a great deal more territory in the future. It is possible that in subsequent studies, the inclusion of numerous APIs may be studied for their potential joint biological benefits against multiple diseases. On the other hand, because nanotechnology encompasses such a broad range of applications, researchers conducting future studies may potentially examine research in which two or more delivery systems coexist to treat a wide variety of illnesses.

While plant-based vesicular delivery systems hold promise for respiratory diseases, addressing the deficiencies in loading capacity, stability, targeting, and scalability, and conducting comprehensive preclinical and clinical evaluations, are crucial for their future development. Exploring combination therapies and optimizing manufacturing processes will also contribute to the advancement and broader application of plant-based vesicular systems in the treatment of respiratory diseases.

Exploring the incorporation of plant-based vesicles into hydrogels or fibers as bio-responsive phytosomes can address the deficiencies of plant-based vesicular delivery systems in respiratory diseases. Through optimization of formulation parameters, in vivo efficacy studies, clinical translation, and patient-specific tailoring, the potential of these systems can be further explored, leading to the development of effective and targeted therapeutic approaches for respiratory diseases.

## Figures and Tables

**Table 1:** Summary of structural properties that make plant-derived compounds potentially beneficial for pulmonary health.

Compound	Structure	Significant Structural Qualities	Ref.
Curcumin	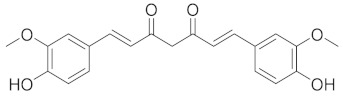	The aromatic rings linked by a chain of carbonyl groups enable curcumin to act as a potent antioxidant.	[73]
Allicin	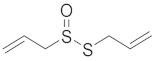	Allicin organosulfur compounds are associated with various health benefits, including antimicrobial, anti-inflammatory, and antioxidant properties.	[74]
Quercetin	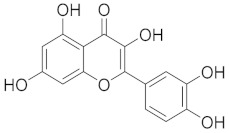	This structural arrangement of aromatic rings allows quercetin to interact with various enzymes, receptors, and cellular components involved in respiratory health.	[75]
Embellin	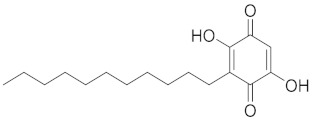	The ring with conjugated carbonyl group has antioxidant and anti-inflammatory properties. Quinones can take electrons and engage in redox reactions, making them potentially useful in cells.	[76]
Berberine	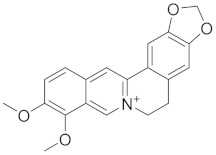	The tetracyclic structure with isoquinoline and benzyl rings allows berberine to interact with different cellular targets and affect respiratory health pathways.	[77]
Gingerol	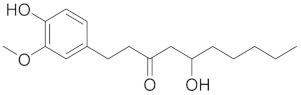	Gingerol has antioxidant, anti-inflammatory, and antibacterial effects due to its structure that is characterized by the presence of a phenol group attached to an aromatic ring.	[78]
Epigallocatechin gallate (EGCG)	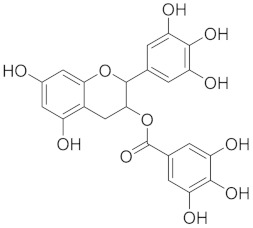	EGCG interacts with various cellular targets and respiratory health pathways due to its flavan-3-ol backbone’s hydroxyl group configuration.	[72]
Resveratrol	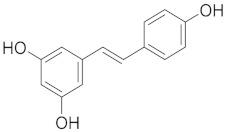	The specific arrangement of aromatic rings and a double bond gives resveratrol antioxidant, anti-inflammatory, and antibacterial effects.	[79]

**Table 2:** Summary of the properties of vesicular drug delivery systems.

VDDS	Description	Advantage	Material Composition	Refs.
Phytosome	Novel phospholipid-bound plant-derived drug delivery formulation that provides an envelope-like coating for the pharmaceutical ingredient; the main component of herbal extracts remains protected from degradation by digestive secretions and bacteria.	Phytosomal drug delivery systems have distinctive attributes, viz., small size, which enables phytocomponents to reach the target site or receptor by passing through the vesicular membrane of phytosomes, and physiochemical properties that include amphiphilicity, biocompatibility, and potential for targeted and controlled payload release.	Consists of the interaction between phytoconstituent functional groups and the polar groups in phospholipid molecules. Contains the active ingredient attached to the polar head of the phospholipid, being an essential element of the micellar membrane.	[134,135,136,137,138,139]
Liposome	Colloidal drug delivery device with a lipid layer surrounding an aqueous center. Drug distribution depends on solubility in the hydrophilic core or lipid layer.	Because of its excellent biocompatibility, simplicity of manufacture, and chemical diversity, molecules that are hydrophilic, amphiphilic, and lipophilic can be loaded.	Drugs having a higher lipophilicity are found in the lipid layer, while those with a higher water solubility reside in the core.	[129,140,141,142,143,144]
Transferosome	An artificial vesicle called a transferosome carrier is made to resemble a cell vesicle or a cell that is exocytosing, making it appropriate for regulated and possibly targeted drug administration.	When applied non-occlusively, transferosome increase transdermal medication delivery. Squeezing along the stratum corneum’s inter-cellular sealing lipid helps transferosomes permeate. They distribute low- and high-molecular-weight medicines.	Transferosomes have a complex lipid bilayer around an aqueous center. The bilayer’s shape and local composition make the vesicle self-regulating and optimal.	[122,145,146,147,148,149,150]
Niosome	Multilamellar vesicular structure of nonionic surfactants, comparable to liposomes, but without phospholipids. Hydrophilic medications can be encapsulated in the core aqueous compartment and hydrophobic drugs in the lipid membrane.	The entrapping of a greater number of compounds, a greater degree of stability, the elimination of the requirement for special handling or storage conditions, as well as the accessibility and low cost of produced materials are all advantages of this method.	Includes the nonionic surfactant as well as the additives as its two constituent parts. The vesicular layer is made up of the non-ionic surfactants. Additives that are utilized in the creation of the niosomes are cholesterol and charged molecules.	[123,151,152,153,154,155]
Pharmacosome	These lipid-based drug delivery systems utilize colloidal dispersions of covalent, amphiphilic medicinal agents to facilitate the translocation of drugs across membranes, tissues, and cellular barriers within the organism.	Amphiphilic substances dissolve better in GI fluid and absorb better via lipophilic membranes. This enhances medication absorption. Complexing both types of drugs improve biological characteristics.	Has positive and negative charges, water- and fat-loving characteristics, and an optimal polyphenol-phospholipid complex ratio.	[136,155,156,157,158]
Ethosome	Ethosomes are delivery carriers that are soft, pliable, and non-invasive. They improve the delivery of active medicines to the systemic circulation by enhancing the distribution of these agents.	Ethosomes are capable of completely penetrating the skin, which allows them to significantly improve the transport of drugs via the skin. Ethosomes also have a high degree of deformability and trapping efficiency.	Ethanol, phospholipids, and water are the three primary components of an ethosome. Ethanol is known to disrupt the organization of the lipid bilayers in the skin, the high quantity of ethanol found in the ethosomes gives them a distinctive quality.	[124,159]

**Table 3:** Summary of the work done on plant-derived APIs incorporated into VDDS for various pulmonary diseases.

VDDS	Encapsulated Plant Material	Critical Quality Attributes (CQA)	Application	Outcomes	Ref.
Phytosomes	Naringenin	ZP = 20.97 ± 0.55 mV PS = 150.8 ± 6.9 nm %EE = 92.1% ± 1.87%	Lung injury	A significant reduction in cytokine expression was observed with naringenin-loaded DPPC phytosomes for dry powder inhalation (NPDPIs) in the treatment of pulmonary edemas.	[231]
Phytosome	Gingerol	ZP = −13.11 mV PS = 254.01 ± 0.05 nm %EE = 86.02 ± 0.18%	Respiratory infection	Pharmacodynamic parameters showed a sustained antibacterial action as well as considerable anti-inflammatory effects against both Gram-positive and Gram-negative bacteria responsible for respiratory infections.	[232]
Phytosome	*Boswellia serrata*		Asthma	Patients receiving phytosome needed fewer inhalations. Insomnia and nausea were the only mild-moderate adverse events associated with phytosome treatment.	[233]
Liposome	Curcumin	ZP = −61.0 mV PS = 271.3 ± 3.06 nm %EE = 81.1%	Asthma	Curcumin liposome formulation suppressed pro-inflammatory markers (IL-6, IL-8, IL-1β, and TNF-α) in BCi-NS1.1 cell line.	[234]
Liposome	a-tocopherol		Lung injury	It has been demonstrated that liposomal a-tocopherol formulation is highly effective at preventing oxidative lung damage.	[235]
Liposome	Paclitaxel		Lung cancer	Decreased number of observable tumor foci on the lung surfaces, increased medication effectiveness, and longer survival.	[236]

## Data Availability

No new data were created or analyzed in this study. Data sharing is not applicable to this article.
